# Physiological, perceived, and physical demands of recreational 3 × 3 basketball and high-intensity interval training in sedentary adult women

**DOI:** 10.5114/biolsport.2025.148547

**Published:** 2025-04-14

**Authors:** Berk Golluceli, Sigitas Kamandulis, Marco Pernigoni, Inga Lukonaitiene, Rasa Kreivyte, Daniele Conte

**Affiliations:** 1 Department of Coaching Science, Lithuanian Sports University, 44221 Kaunas Lithuania; 2 Institute of Sport Science and Innovations, Lithuanian Sports University, 44221 Kaunas, Lithuania; 3 Department of Movement, Human and Health Sciences, University of Rome “Foro Italico”, 00135 Rome, Italy

**Keywords:** Sedentary behaviour, Physical activity, Health, Team sports, Women sport

## Abstract

The aim of this investigation was to compare the acute physiological responses – percentage of maximal peak heart rate (%HRpeak) and blood lactate (BLa) – as well as perceived demands (rating of perceived exertion, RPE) and enjoyment of a recreational 3 × 3 basketball match and high-intensity interval training (HIIT) in sedentary women. Twelve healthy, adult sedentary women (age: 37 ± 14 years; body mass: 66 ± 19 kg; stature: 162 ± 13 cm; fat mass: 27.5 ± 12.5%) performed a 3 × 3 basketball match and HIIT including gym-based activities with comparable duration. The %HRpeak was continuously monitored during, the BLa was assessed before and after, and the RPE and enjoyment were collected at the end of each protocol. 3 × 3 basketball elicited higher %HRpeak (p < 0.001; d = -1.64, large). BLa analysis demonstrated a significant difference for all the between- and within-condition comparisons (p < 0.05; r-values: large) except for the comparison between pre-HIIT and pre-3 × 3 basketball match (p > 0.05; r-value: 0.052, no effect) and between post-HIIT and post-3 × 3 basketball match (p = 0.072; r-value: 0.495, medium). Similar low RPE (4 ±1 AU; p = 0.999; r-value = < 0.001, no effect) and high enjoyment (3 × 3 basketball = 6 ± 1 AU; HIIT = 5AU ± 1 AU, p = 0.233; r-value = 0.250, small) values were found between conditions. 3 × 3 basketball induced overall higher %HRpeak compared to HIIT, suggesting its suitability as a health-enhancing activity for sedentary adult women. Moreover, since both conditions highlighted low RPE and high enjoyment values, they have the potential to be effective in enhancing the training adherence in sedentary adult women.

## INTRODUCTION

Regular physical activity (PA) has been identified as one of the main factors able to lower the mortality risk and risk for chronic diseases while enabling primary disease prevention in the adult population [1]. Conversely, physical inactivity and sedentary behaviour are considered among the main public health concerns since they are associated with adverse effects on health such as heart disease, diabetes, pulmonary function, and joint stiffness [2, 3, 4]. This phenomenon is particularly evident in adult women, who have been shown to be less physically active than their male counterpart [5, 6, 7, 8, 9]. For instance, an international survey conducted across 168 countries demonstrated a higher inactivity rate in adult women compared to men (31.7% vs. 23.4%, respectively) [8]. Therefore, it seems necessary for researchers and practitioners to find suitable training interventions able to close the gender gap and allowing women adult to take part in properly designed physical activity programmes.

High-intensity interval training (HIIT) is a training method that has been extensively studied for its beneficial effects on fitness level and various health markers of the general population [10, 11, 12]. This training typology is usually characterized by short duration with various work-to-rest ratios, and it has been shown to stimulate physiological adaptations similar to or greater than those produced by longer-duration traditional aerobic exercises such as running or cycling [13]. When considering the women population, a meta-analysis showed that > 8 weeks of HIIT training was able to provide beneficial effects in sedentary adult women, such as weight loss and reduction in adipose tissue [14], making HIIT a successful training strategy in this population. However, the appropriateness of this training method for the sedentary population is still debated in the scientific community. Indeed, while Reljic et al. [15] demonstrated a low (i.e., 13.4%) dropout rate of participants enrolled in HIIT trials lasting ≥ 4 weeks, it has been suggested [16] that HIIT can be perceived as too strenuous for the sedentary population. Overall, it is possible that some participants will not be willing to engage in typical HIIT activities, which can be perceived as too hard and can be associated with negative feelings. Therefore, it is important for sport and health practitioners to design and implement alternative training methods willing to increase sedentary participants’ acceptance and training adherence.

Team sports such as football, handball, or basketball are alternative training methods aimed at increasing adults’ physical activity levels [17, 18, 19]. In particular, basketball activities have been considered a potentially valid alternative to other training methodologies in improving participants’ health status [17, 20, 21, 22]. Indeed, a recent study [20, 21] comparing the acute effect of 3 × 3 basketball games and HIIT including gym-based activities in young male adults found that basketball activities elicited higher physiological demands (percentage of maximal heart rate) and enjoyment, and a lower blood lactate and rating of perceived exertion (RPE) compared to HIIT. However, these results referred only to active young male adults regularly playing basketball, while the physiological and perceived response and the enjoyment levels might differ in the sedentary population with little basketball experience. Potentially, sedentary people might not have a real preference in terms of enjoyment for any of those activities, which are both characterized by a short, high-intensity session. However, no prior research has compared the acute physiological and perceived demands of a recreational 3 × 3 basketball match and a HIIT session in sedentary adult women. This comparison is warranted to have a better understanding of whether basketball activities can have a response similar to that for HIIT, which was proven to have a positive effect on sedentary women’s health status [23]. Additionally, it should be noted that no previous investigation has assessed the physical demands encountered by sedentary people participating in a 3 × 3 basketball match. This missing information seems essential for sport and health practitioners to fully understand whether 3 × 3 basketball matches can produce a proper external load (accelerations, decelerations, etc.) able to elicit physiological and perceived responses positively impacting on the health status of sedentary people. Therefore, the aims of this study were: a) to compare the physiological and perceived demands of HIIT and a 3 × 3 basketball match, and b) to assess the physical load characterizing the 3 × 3 basketball match in sedentary adult women.

## MATERIALS AND METHODS

### Participants

Twelve apparently healthy sedentary adult women (age: 37 ± 14 years; body mass: 66 ± 19 kg; stature: 162 ± 13 cm; fat mass: 27.5 ± 12.5%) volunteered to participate in this study. Participants were mainly students and staff of the university in which the study was conducted and were contacted by email about one month before the commencement of the research procedures. An a priori analysis indicated that the present study is sufficiently powered (minimum sample size = 8) using α = 0.05, β = 0.95 and an effect size = 1.6 (G*Power, version 3.1.9.2; University of Dusseldorf; Germany) based on the difference in %HRpeak calculated between recreational 3 × 3 basketball and HIIT in young male adults [17]. Prior to the commencement of the study, participants completed the Physical Activity Readiness Questionnaire (PAR-Q) to rule out any health conditions that might have prevented them from taking part in the current investigation. Only participants reporting no acute pain, history of cardiovascular disease, muscle relaxant medications and/or any type of injury within the last 6 months before the commencement of the study were included. Furthermore, participants filled in a specifically designed questionnaire encompassing questions about the reported weekly physical activity levels in accordance with the guidelines set by the World Health Organization [24], which categorized participants as sedentary, physically inactive. Participants with any basketball-specific or HIIT-specific skill levels were allowed to participate in the study. Finally, participants sign their informed consent after being informed of the procedures, advantages, and dangers associated with participation in the study. The procedures received approval by the ethics committee of the Lithuanian Sports University Ethics Committee [approval number: MNL-SVA (M)- 2023-546]

### Study design

**FIG. 1:**
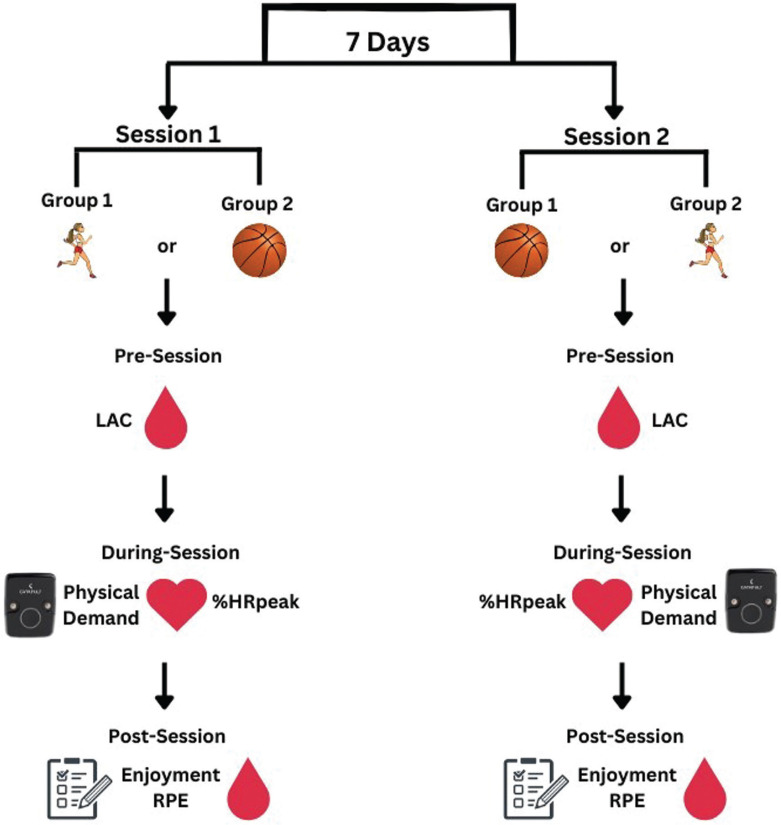
Illustration of the full study design from Day 1 to Day 8. Note: = high-intensity interval training; = recreational 3 × 3 basketball. = lactate collection; = monitoring physical load (only during 3 × 3 basketball matches); = monitoring heart rate; %HRpeak = percentage of peak heart rate; = assessment of enjoyment and rating of perceived exertion (RPE)

The acute physiological, perceived, and physical demands of recreational 3 × 3 basketball matches and HIIT sessions were evaluated using a descriptive design across two experimental sessions (Figure 1) following a familiarization session. Stature, body mass, and percentage of fat mass (%fat mass) were collected in the familiarization session. Moreover, after receiving thorough explanations from the research staff members about the adopted scales and all other procedures, participants were instructed to undergo 5 min of both recreational 3 × 3 basketball match simulation and HIIT sessions to become familiar with the study protocols.

After the familiarization session, participants were randomly and equally divided into two groups. Participants completed either a recreational 3 × 3 basketball match or HIIT in two separate experimental sessions, separated by one week. To prevent the potential effects of circadian rhythms, all experimental sessions were arranged at the same time of day (group 1: 8.30 am–10.00 am; group 2: 10.00 am–11.30 am). Additionally, participants were instructed to maintain regular sleeping and eating schedules, refrain from caffeine and alcohol prior to each experimental day, and engage in no physical activity for the 48 hours prior to and after the recreational 3 × 3 basketball match or HIIT session.

### Procedure

The HIIT sessions and the 3 × 3 basketball matches were performed on an indoor regular-sized wooden basketball court and preceded by a standardized 10-min warm-up consisting of jogging, dynamic stretching and basketball drills including dribbling and shooting skills. An HIIT typology including gym-based activities was adopted, which was previously used in adult populations [25, 17]. This HIIT modality encompasses a 12-min duration and a work-to-rest ratio of 1:1 with 30-s bouts (Bout 1: 4 × push-ups + 10 m shuttle sprint; Bout 2: 4 × squat jump + 10 m shuttle side-step; Bout 3: 4 × sit-ups + 10 × jumping jacks) interspersed by 30-s rest periods for a total of 4 sets [25, 17]. In each bout, participants were required to complete as many rounds of activities as possible. The 3 × 3 basketball match was played on a half basketball court with one basket and ended either after 10 min of match play, or when one of the two teams scored 21 points as defined in the 3 × 3 basketball rules for official competition. For the matches played, none of the involved teams reached 21 points with matches ending after 10 min of match play. Moreover, a 12-second shot clock and the official ball (size 6) used in 3 × 3 basketball competitions were employed. Each team was composed of three players randomly selected by the research group. No tactical information was provided for the participants, while they were instructed to play following the prescribed rules. Both HIIT and 3 × 3 basketball sessions were supervised by the members of the research staff with constant verbal encouragement provided in both activities.

Participants’ heart rate (HR) was continuously recorded during the HIIT sessions and 3 × 3 basketball matches using Bluetooth chest belts (H10, Polar Electro Oy, Kempele, Finland). Each belt was connected to participants’ smartphone through the Polar Beat app, which has been previously used [26]. At the end of each activity, data were transferred onto researchers’ Polar cloud account, and successively downloaded to Microsoft Excel spreadsheets for further analyses. The peak HR was identified as the highest HR value recorded across the HIIT session and/or 3 × 3 basketball match. Then, HR values were expressed as the percentage of the peak HR (%HRpeak) for each activity. Moreover, blood samples were collected from the ear lobe before (in a rested condition) and after (at 1 min and 5 min) each HIIT session and 3 × 3 basketball match to assess the blood lactate concentration. The peak blood lactate value was identified as the highest value of the two post-session samples. The Lactate Pro 2 CT-1730 analyser (Arkray Co., Kyoto, Japan) was used to examine blood samples [27].

At the conclusion of each protocol, each participant’s rating of perceived exertion (RPE) was collected using the modified Borg 10-point RPE scale (CR-10) [28]. Furthermore, the Exercise Enjoyment Scale (EES) was used to assess participants’ enjoyment responding to the statement: “Use the following scale to indicate how much you are enjoying this exercise session” through a 7-point Likert scale (1 – “not at all”; 2 – “very little”; 3 – “slightly”; 4 – “moderately”; 5 – “quite a bit”; 6 – “very much”; 7 – “extremely”) [29]. Both scales were previously used to assess RPE and enjoyment in HIIT and 3 × 3 basketball matches, respectively [17].

Prior to the 3 × 3 basketball matches, participants were individually equipped with inertial measurement units (IMU) (Catapult Clear-Sky T6, Catapult Innovations, Melbourne, VIC, Australia) placed in manufacturer-provided vests for secure attachment onto each player between the scapulae. Through IMU by checking and saving necessary data, time and stoppage time throughout the session day, the PlayerLoad (PL) and its value calculated per minute (PL/min), the number of accelerations (ACC), decelerations (DEC), changes of direction (COD), and jumps (JUMP) were collected and further downloaded to the Catapult proprietary software (Catapult Openfield, v1.18, Catapults Innovations, Melbourne, VIC, Australia) for further analysis. These load measures are widely used in basketball research to assess the physical demand in professional and non-professional basketball players and in the general populations such as young and older adults [30; 17, 31]. IMU devices were not used to assess the physical demand of the HIIT sessions since they include gym-based static exercises such as push-ups and sit-ups, which cannot be correctly detected by accelerometers [17]. IMU has been suggested to be more useful to assess the physical demand for recreational team sport activities such as basketball [17].

### Statistical analysis

Mean and standard deviation (SD), median and interquartile range (IQR) and percentages (%) were calculated as descriptive statistics. Only descriptive analysis was performed for physical demand measures, since they were collected only for the 3 × 3 basketball matches. Inferential statistics were used for all other dependent variables. The Shapiro-Wilk test was used to determine the normality of the data for each continuous dependent variable, and it showed that the data for blood lactate were not normally distributed. Therefore, a paired-sample t-test was used to compare the differences in %HRpeak between the recreational 3 × 3 basketball and HIIT conditions. A non-parametric approach was applied to the ordinal (i.e., RPE and enjoyment) and non-normally distributed (i.e. blood lactate) variables. To measure the variations in RPE and enjoyment between recreational 3 × 3 basketball and HIIT conditions, a Wilcoxon signed-rank test was used. Additionally, a Wilcoxon signed-rank test with Bonferroni corrections was utilized to evaluate the variations in blood lactate between and within conditions. For data analysed via parametric statistics, Cohen’s d was used as a measure of the effect size, and values were interpreted using Hopkins’ benchmarks as follows: trivial < 0.20, small 0.2–0.59, medium 0.60–1.19, large 1.20–1.99, and extremely large ≥ 2.0 [32]. Lastly, the r value (Z/√N) was chosen as the effect size for non-parametric statistics, and it was interpreted using Cohen’s benchmarks classifying 0.1, 0.3, and 0.5 as small, medium, and large effect sizes, respectively [33] The Jamovi software for Windows (version: 2.2.5; retrieved from https://www.jamovi.org) was used for the statistical analyses and the significance level was set at 0.05.

## RESULTS

**FIG. 2:**
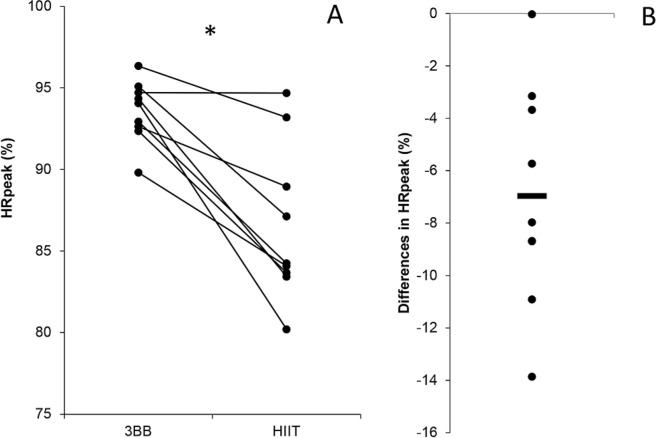
Individual differences in peak heart rate (%HRpeak) between recreational 3 × 3 basketball (3BB) and high-intensity interval training (HIIT) (**A**). Mean difference in %HRpeak between 3BB and HIIT (**B**). Note: * = indicates p < 0.001.

A higher %HRpeak (HIIT = 86.6% ± 4.8 and 3 × 3 basketball = 93.6% ± 1.9; p < 0.001; mean difference (95%CI) = 6.97 (3.69; 10.2); Cohen’s d (95%CI) = 1.64 (0.59; 2.64), large) was found for the 3 × 3 basketball match compared to the HIIT session (Figure 2).

**FIG. 3:**
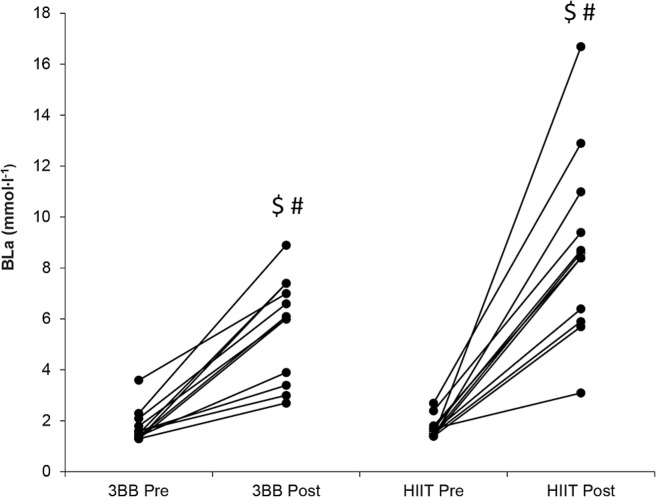
Individual changes of blood lactate concentrations (Bla) in recreational 3 × 3 basketball match (3 × 3BB) and high-intensity interval training (HIIT) at different time points (i.e. pre- and post-). # indicates difference compared to pre-HIIT (p < 0.05); $ = indicates difference compared to pre-3 × 3BB (p < 0.05).

The lactate analysis demonstrated a significant difference throughout all the between- and within-condition comparisons (p < 0.05; r-values: large) except for the comparison between pre-HIIT and pre- 3 × 3 basketball match (p > 0.05; r-value: 0.052, no effect) and between post-HIIT and post-3 × 3 basketball match (p = 0.072; rvalue: 0.495, medium) (Figure 3). The descriptive statistics analysis showed the following median ± IQR values: pre-HIIT = 1.6 ± 0.3 mmol∙l^−1^; post-HIIT: 8.5 ± 3.5 mmol∙l^−1^; pre-3 × 3 basketball = 1.6 ± 0.5 mmol∙l^−1^; post-3 × 3 basketball = 6.1 ± 3.3 mmol∙l^−1^.

**FIG. 4:**
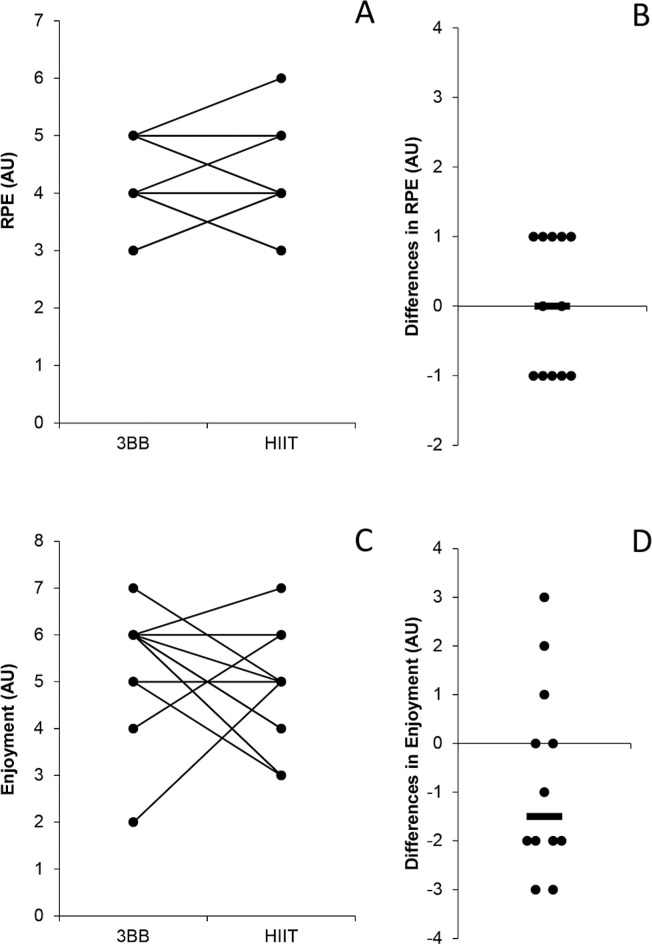
Individual differences in rating of perceived exertion (RPE) (**A**) with median differences (**B**) and enjoyment (**C**) with median differences (**D**) between recreational 3 × 3 basketball (3BB) and high-intensity interval training (HIIT). Note: AU = arbitrary units.

No significant difference was found in RPE (median ± IQR for HIIT = 4 ± 1 AU and 3 × 3 basketball = 4 ± 1 AU; p = 0.999; rvalue = < 0.001, no effect) and enjoyment (median ± IQR for HIIT = 5 ± 2 AU and 3 × 3 basketball = 6 ± 1 AU; p = 0.233; r-value = 0.250, small), between HIIT and the 3 × 3 basketball match (Figure 4). The descriptive statistical analyses of the physical demands showed that the 3 × 3 basketball match led to the following external load results: PL = 76.3 ± 16.6 AU; PL/min = 6.7 ± 1.5 AU/min; ACC = 14.5 ± 5.3; DEC = 18 ± 10.2; COD = 86.5 ± 25.2; JUMPS = 6.1 ± 8.2.

## DISCUSSION

The primary aim of this study was to assess the differences in acute physiological and perceived responses between a recreational 3 × 3 basketball match and HIIT in sedentary adult women. The main findings revealed that recreational 3 × 3 basketball resulted in higher heart rate responses compared to HIIT, while no significant differences were found for blood lactate concentration (although with a medium effect size in the between-condition comparison after completion of the activities), enjoyment and RPE. These results underline the potential of 3 × 3 basketball as recreational activity to generate health benefits in sedentary adult women.

The 3 × 3 basketball match induced an exercise intensity of 93.6 ± 1.9% of the HRpeak, which is slightly higher compared to the 91.2 ± 3.5% of the HRpeak documented in active young male adults [17]. A possible reason for this difference is the different assessment of the HRpeak, since, in the current investigation, we considered the HRpeak as the highest HR value reached during the 3 × 3 basketball match or HIIT, while in a previous study [17], the HRpeak was measured during an incremental test to exhaustion (i.e. 30–15 Intermittent Fitness Test). Possibly, during the incremental test, participants could reach higher HR responses with respect to recreational basketball and/or HIIT, which could explain the higher results recorded in our study. Overall the %HRpeak found in our study during 3 × 3 basketball matches is high and can be considered as vigorous according to the American College of Sports Medicine’s (ACSM) benchmarks (i.e. 77%–95% of maximum heart rate) [34]. Furthermore, the 3 × 3 basketball match elicited a largely higher %HRpeak compared with HIIT, similarly to the results of a previous investigation assessing the same protocols in active young male adults [17]. The different structure of the two considered activities could explain this outcome, since HIIT includes mainly static activities (push-ups, sit-ups, etc.), which might not affect HR as much as the more dynamic actions typical of 3 × 3 basketball. These results overall indicate that 3 × 3 basketball matches can induce higher involvement of the cardiovascular system compared to the applied HIIT modality, providing essential information for sport and health practitioners. In fact, it was previously found that activities with these intensities may facilitate prevention and alleviation of cardiovascular disease mortality, and occurrence of cancer and diabetes in the general population, including sedentary women [24].

The analysis of the anaerobic glycolytic system showed that both the 3 × 3 basketball match and HIIT induced an increased lactate concentration measured at post-activity time points compared with the rest condition. It should also be noted that, while not significant (p = 0.072), higher values (medium effect size; r-value: 0.495) were found in HIIT compared to the 3 × 3 basketball match at the post-activity time point. This outcome is consistent with a previous investigation assessing the blood lactate concentration in active young male adults [17], and it can be explained by the high level of muscular stress experienced by sedentary people in performing upper and lower body strength exercises during HIIT, resulting in a greater level of lactic acidosis [35]. Another interesting point to consider is that the 3 × 3 basketball match elicited mean and median blood lactate concentrations of 5.7 ± 2.0 mmol ∙ l^−1^ and 6.1 ± 3.3 mmol ∙ l^−1^, respectively, similarly to the average values recorded in female 3 × 3 basketball official competitions (~ 6 mmol ∙ l^−1^) [36], suggesting no influence of the participants’ fitness status on this physiological measure. Overall, the 3 × 3 basketball match and HIIT induced a modest level of metabolic stress, suggesting that probably a combination of aerobic and anaerobic systems is mainly involved for these activities.

When considering participants’ perceived exertion, similar RPE values were observed at the end of recreational 3 × 3 basketball and HIIT, with median values of 4 (i.e. *somewhat hard*). The relatively low RPE reported after 3 × 3 basketball was quite expected, as it is comparable with the findings for other team sports, which are usually considered enjoyable activities resulting in low perceived effort [37, 38]. On the other hand, the relatively low RPE values recorded after HIIT are surprising, considering the high muscular demand for the performed exercises (push-ups, sit-ups, etc.), particularly for sedentary people. It could be speculated that the recovery time across the HIIT protocol might mitigate the overall perceived exertion and impact on the cognitive approach to managing the exertion that comes with intense exercise. This idea is reinforced by the results of Kilpatric et al. [39], which showed that performing more intervals of shorter durations (30-s bouts) appears to produce lower post-exercise RPE values (~4 AU) than performing fewer intervals of longer duration (120-s bouts) and equal intensity in overweight sedentary people. Furthermore, it should be noted that, since the activities were performed with a self-selected level of exertion, it is possible that our participants paced themselves across the HIIT and the 3 × 3 basketball matches due to their lack of experience in performing these protocols, diminishing the perceived exertion of both activities.

The lack of significant differences in RPE values between the 3 × 3 basketball match and HIIT is also reflected in the participants’ perceived enjoyment. Interestingly, both 3 × 3 basketball (6 AU – *very much*) and HIIT (5 AU – *quite a bit*) were considered enjoyable, which contrasts with a previous investigation documenting statistically higher enjoyment during 3 × 3 basketball matches (6 AU – *very much*) compared to HIIT (4 AU – *moderately*) in active young adults [17]. A possible explanation for this difference could be the different levels of previous basketball experience and skill of the participants across the two studies (i.e. sedentary women with limited basketball experience and skill vs. active young adults regularly playing basketball at a recreational level). In fact, it has been previously suggested that competency in exercise might be considered as a key contributor to its enjoyment [40]. Therefore, it is possible that participants possessing sport-specific basketball experience might experience lower enjoyment during HIIT than playing basketball, while this difference is less evident in participants without previous basketball experience. Overall, this result suggests that both activities can be considered enjoyable and might have the potential to enhance the training adherence during long-term training programmes in the sedentary population without previous basketball experience.

To the best of our knowledge, this is the first study assessing the physical load performed by sedentary women during 3 × 3 basketball matches, which might represent benchmarking values for sport and health practitioners intending to use 3 × 3 basketball matches as health-enhancing tools in sedentary women. In general, sedentary women registered a mean PL of 76.3 AU, which is considerably lower compared to female athletes competing in official 3 × 3 basketball matches (range: 95.3–128.9 AU) [36], suggesting that our participants performed at low intensity. Moreover, due to different measurement devices and data reduction (absolute values vs. values relative per playing time) it is hard to make a sound comparison of ACC, DEC, COD, and JUMPS values with those reported in previous investigations assessing 3 × 3 basketball female players or other general populations. Nevertheless, we consider the analysis of these physical load measures as fundamental, since they might improve bone health in sedentary women, who are particularly exposed to bone demineralization and osteoporosis [41]. Therefore, future studies are warranted to investigate whether the ACC, DEC, COD and JUMPS elicited during 3 × 3 basketball matches across a long-term period might positively affect bone health in sedentary women.

Although this study provides novel data about the comparison of the physiological and perceptual responses during 3 × 3 basketball matches and HIIT in sedentary women, some limitations should be mentioned. Firstly, our results are limited to adult sedentary women, and other sedentary populations (i.e. young and/or older adults) might produce different results. Secondly, this study assessed only the acute effects of HIIT and 3 × 3 basketball matches, while no information is available on their long-term effect. Therefore, future investigations should implement intervention designs addressing the physiological and perceptual responses of these training modalities over time in various general populations.

## CONCLUSIONS

The results of the study showed that 3 × 3 basketball matches elicit high %HRpeak values compared to HIIT, suggesting that it might be considered a valuable sport activity to increase the health of sedentary adult women from a cardiovascular standpoint. Moreover, considering the low RPE and high enjoyment values found in both conditions, it can be concluded that both 3 × 3 basketball matches and HIIT have the potential to generate high training adherence over time in sedentary adult women. Finally, the physical load induced by 3 × 3 basketball was overall at low intensity, providing reference values for sport and health practitioners.
